# Biomimetic Elastomer–Clay Nanocomposite Hydrogels with Control of Biological Chemicals for Soft Tissue Engineering and Wound Healing

**DOI:** 10.1021/acsabm.4c01944

**Published:** 2025-02-20

**Authors:** Sungkwon Yoon, Biqiong Chen

**Affiliations:** a School of Mechanical and Aerospace Engineering, Queen’s University Belfast, Stranmillis Road, Belfast BT9 5AH, United Kingdom; b Department of Materials Science and Engineering, University of Sheffield, Mappin Street, Sheffield S1 3JD, United Kingdom

**Keywords:** polymer nanocomposites, poly(glycerol sebacate), soft tissue engineering, wound healing, malodorous diamines

## Abstract

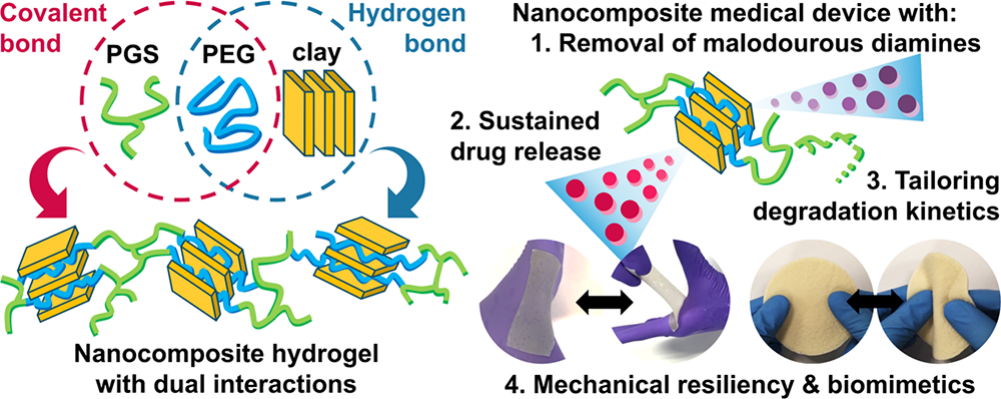

Resilient hydrogels are of great interest in soft tissue applications, such as soft tissue engineering and wound healing, with their biomimetic mechanical and hydration properties. A critical aspect in designing hydrogels for healthcare is their functionalities to control the surrounding biological environments to optimize the healing process. Herein, we have created an elastomer–clay nanocomposite hydrogel system with biomimetic mechanical behavior and sustained drug delivery of bioactive components and malodorous diamine-controlling properties. These hydrogels were prepared by a combined approach of melt intercalation of poly(ethylene glycol) and montmorillonite clay, followed by *in situ* cross-linking with a branched poly(glycerol sebacate) prepolymer. The hydration, vapor transmission, and surface wettability of the hydrogels were readily controlled by varying the clay content. Their mechanical properties were also modulated to mimic the Young’s moduli (ranging between 12.6 and 105.2 kPa), as well as good flexibility and stretchability of soft tissues. A porous scaffold with interconnected pore structures as well as full and instant shape recovery was fabricated from a selected nanocomposite to demonstrate its potential applications as soft tissue scaffolds and wound healing materials. Biodegradability and biocompatibility were tested *in vitro*, showing controllable degradation kinetics with clay and no evidence of cytotoxicity. With the high surface area and absorption capacity of the clay, sustained drug delivery of a proangiogenic agent of 17β-estradiol as a model drug and the ability to control the malodorous diamines were both achieved. This elastomer–clay nanocomposite hydrogel system with a three-dimensional interconnected porous scaffold architecture and controllable hydration, mechanical, and biodegradable properties, as well as good biocompatibility and the ability to control the biological chemical species of the surrounding environments, has great potential in soft tissue engineering and wound healing.

## 1 Introduction

Soft body tissues are essentially hierarchically and sophisticatedly structured multifunctional hydrogels. Cells in soft body tissues signal growth factors, produce the extracellular matrix, and eventually build complex tissues around them, which are mediated by aqueous body fluids.^1,2^ Regenerative medicine is biomimetic in its nature, aiming to mimic these cellular activities to regenerate new tissues to cure traumatic injuries and damages of tissues.^3^

In this regard, resilient hydrogels pose several advantages as soft tissue engineering scaffolds and wound healing materials, with their biomimetic soft mechanical behavior and hydration capacity that allow the diffusion of desired chemical and biological species.^4^ A critical aspect in designing these advanced biomaterials is their ability to control the surrounding biological environment to optimize the tissue regeneration and the wound healing processes. This can be done by controlled delivery, or sometimes removal, of bioactive chemical species at the target tissue or trauma sites. For instance, angiogenesis, growth of new blood vessels from existing vessels, has a crucial impact on tissue regeneration. Regeneration of skin tissues essentially depends on the angiogenesis as a highly vascularized tissue.^5^ In wound healing, it was found that angiogenesis governs the healing rate.^6^ Angiogenesis is typically achieved with delivery of proangiogenic agents to the target tissues, such as heparin, vascular endothelial growth factor, platelet-derived growth factor, fibroblast growth factor basic, and 17β-estradiol (E2).^7,8^ Delivery of these proangiogenic agents needs to be controlled to have optimized angiogenesis. For instance, heparin shows concentration-dependent angiogenic and antiangiogenic behaviors.^9^

Another functionality that could be beneficial for optimizing the soft tissue engineering and the wound healing processes is the control over malodorous diamine compounds such as putrescene (PUT) and cadaverine (CAD). These diamine compounds are found commonly in chronic wound sites such as diabetic or cancerous necrotic wounds, mainly due to the decomposition of wound tissues by anaerobic bacteria.^10^ They are known to cause delayed or incomplete healing processes by impeding cell migration, inhibiting tissue transglutaminase, or infection.^10−12^ They are also a significant cause of discomfort in patients during medical practice.^10^

Poly(glycerol sebacate) (PGS) is a synthetic polyester elastomer.^13^ It has been greatly explored in various healthcare applications such as soft tissue engineering, drug delivery, and surgical devices due to its biomimetic and controllable mechanical behaviors, proven biocompatibility, and nontoxic degradation products.^14^ However, hydrophobicity of PGS with limited water swelling capacity (2–5%) requires modification on the polymer to fabricate hydrogels.^13,15^ This is usually done by copolymerization of PGS with a hydrophilic polymer segment such as poly(ethylene glycol) (PEG)^16^ or gelatin.^15^ However, research in these PGS-based copolymer hydrogels mostly focused on modulating the mechanical properties, hydration behavior, and biodegradability by varying the ratio between the copolymer segments, with no additional bioactive functionalities controlling the surrounding biological environments, such as the delivery and removal of biological chemicals to provide the optimal tissue regeneration. There has been a recent study on angiogenesis with PGS modified by oxidized hyaluronic acid.^17^ However, the study used PGS to produce nanospheres within an injectable hydrogel without exploring the biomimetic mechanical properties of PGS.

Clay minerals have a long history of being used in medical practice. The interest in utilizing these layered silicates for biomedical applications is owing to their colloidal particle size and shape, charged surfaces, high specific surface area, swelling capacity, and cation exchange capability, as well as low cytotoxicity.^18^ Various clay minerals including kaolinite, halloysite, laponite, and montmorillonite (MMT) have been studied in healthcare applications such as tissue engineering, drug delivery, wound healing, and biosensors and actuators.^18^ Recently, our group reported a PGS-based polyurethane–clay nanocomposite hydrogel system with an organically modified MMT for enhanced mechanical properties, as well as controllable hydration, degradation, and drug delivery behavior.^19^

Herein, a new polymer–clay nanocomposite hydrogel system has been created with attractive bioactive functionalities of resilient and biomimetic mechanical behaviors, sustained drug delivery, and malodorous diamine-controlling properties.^20^ Both hydrophilic PEG and sodium MMT are introduced to a PGS prepolymer to produce PGS-based nanocomposite hydrogels. Our hypothesis was that PGS would provide biomimetic and resilient mechanical behaviors as well as biodegradability. PEG was chosen to introduce the hydration property and facilitate the dispersion of sodium MMT within the polymer network through intercalation. MMT modulated the biomimetic mechanical property and provided a sustained drug release and malodorous diamine-controlling behavior via its high surface area and excellent absorption capacity. A combined approach was introduced to prepare polymer–clay nanocomposite hydrogels. PEG was melt-intercalated into MMT galleries, followed by direct *in situ* cross-linking with the PGS prepolymer, utilizing its branched structure without any additional catalysts or cross-linking agents. The resulting cross-linked polymer network structures with dispersed MMT nanoplatelets were saturated in water, yielding a polymer–clay nanocomposite hydrogel. The chemical structure, the dispersion of MMT, the water swelling behavior, and the vapor permeability of the nanocomposites were characterized. The controllable and biomimetic mechanical properties were demonstrated on both the hydrogel samples and their proof-of-concept porous scaffolds. Biodegradability and biocompatibility were evaluated *in vitro*. A model drug, E2, was chosen for its proangiogenic activity for sustained drug release in this new hydrogel system. The capacity of controlling malodorous diamines was tested with PUT and CAD. The benefits and potential of this new nanocomposite hydrogel system in soft tissue engineering and wound healing were discussed based on its material structure and properties.

## 2 Materials and Methods

### 2.1 Materials

Glycerol, sebacic acid, PEG (with a number-average molecular weight of 2000 g mol^–1^), E2, PUT, CAD, mercaptoacetic acid (MAA), *o*-phthalaldehyde (OPA), methanol, ethanol, phosphate-buffered saline (PBS) tablets, lipase from the porcine pancreas (54 U mg^–1^), Dulbecco’s modified Eagle’s medium (DMEM) with high glucose, and resazurin sodium salt were purchased from Sigma-Aldrich.

A sodium MMT clay was provided by Southern Clay Products, purified to remove excessive sodium cations, and dried before use according to a previously reported method (Supporting Information). Ultrapure water (resistivity: 17.1 ± 1.0 MΩ cm at 25 °C) from a Barnstead Smart2Pure system was used.

### 2.2 Synthesis of a PGS Prepolymer

The PGS prepolymer was synthesized following a previously reported polycondensation approach.^13,15^ Glycerol (16.2 mmol) and sebacic acid (26.0 mmol) were charged into a three-neck flask. The monomers were first reacted at 130 °C for 1 h under a flow of nitrogen with a Dean–Stark apparatus and then for another 2 h with a vacuum drying line to remove water vapor during the reaction.

### 2.3 Preparation of Nanocomposite Hydrogels

The purified MMT (0, 5, 10, and 15 parts per hundred rubber, phr, with respect to the total amount of raw materials in the dried nanocomposites) was mixed into a molten PEG (1.6 mmol) at 80 °C in an oil bath by mechanical agitation for 15 h under a nitrogen atmosphere until homogeneous PEG/MMT mixtures were acquired. The PEG/MMT mixture with 15 phr of MMT showed visible solid agglomerates and inhomogeneity and hence not studied further.

Subsequently, the PGS prepolymer previously prepared was added into the PEG/MMT mixtures, the temperature was raised to 130 °C, and the polycondensation reaction was kept for 72 h. A Dean–Stark apparatus with a flow of nitrogen was used for the initial 2 h, followed by drying with a vacuum line for the later 70 h. Highly viscous resins were acquired and collected onto a polytetrafluoroethylene mold. The resins were cohesive and unable to spread evenly onto the mold. A nonstick baking paper (food-grade; purchased from a local store; usable temperature up to 220 °C) was topped, and a 10 N force was applied by a weight to achieve even spreads. Following degassing at 80 °C for 30 min in a vacuum oven to remove voids, curing was performed at 130 °C for 32 h in a vacuum oven, resulting in solid elastomeric nanocomposite films.

After curing, the films were subjected to purification by extraction with a series of water/ethanol solutions (0, 30, 50, 70, and 100%; 12 h each) with agitation at 37 °C on a plate shaker (Cole-Parmer) to get rid of any uncross-linked organic oligomers and excessive MMT. The washed films were then dried for 48 h in a vacuum oven at 37 °C. Finally, the samples were immersed in PBS until saturation (72 h) to prepare swollen nanocomposite hydrogels (NCHs).

### 2.4 Material Characterization

Attenuated total reflectance (ATR) Fourier transform infrared spectroscopy (FTIR) was performed on a PerkinElmer Spectrum One NTS analyzer (500–4000 cm^–1^, resolution: 2 cm^–1^, and number of scans: 16). A pressure of 70 N was applied on the specimens by a built-in screw to extend the degree of sample contact on the diamond ATR crystal.

Gel permeation chromatography (GPC) was carried out on an Agilent Technologies 1260 Infinity. The eluent was tetrahydrofuran containing 2.0% (v/v) triethylamine and a 0.05% (w/v) butylated hydroxytoluene inhibitor. The calibration was done using poly(methyl methacrylate) standards. Samples were filtered twice prior to tests using 0.45 μm PTFE syringe filters.

X-ray diffraction (XRD) patterns were acquired on a STOE STAPI P instrument (Cu Kα_1_, λ = 0.15406 nm, 40 kV, 35 mA, and scan rate: 0.1° s^–1^). The samples were dried in a vacuum oven at 37 °C until constant weights were read and cryo-milled by an SPEX Certiprep 6850 cryogenic mill prior to the test.

Transmission electron microscopy (TEM) was performed on an FEI Tecnai T-12 at an operation voltage of 80 kV. Dry nanocomposite films were cut into a small size and sectioned by a Leica UC6/FC6 cryo-ultramicrotome at −100 °C using a Diatome cryo-P 35 diamond blade. Specimens were placed onto bare 300-mesh copper grids, and images were recorded using a Gatan Orius bottom-mounted camera and Gatan digital micrograph.

Scanning electron microscopy (SEM) was conducted on a Hitachi FlexSEM 1000 at an accelerating voltage of 10 kV. The dry nanocomposites were cut into small pieces, mounted onto an aluminum stub by applying a Pelco conductive silver paste (Ted Pella, USA), and gold-coated by a high-resolution polaron sputter coater (Emscope SC500A).

The water swelling ratio was recorded gravimetrically. Disk-shaped dry nanocomposite specimens (*n* = 3, diameter: 5.3 mm, and thickness: 0.71–0.88 mm) were prepared by a punching utensil and kept in a vacuum oven at 37 °C until constant weights were read. The initial dry weight (*W*_dry_) was then obtained by a four-decimal scale (Sartorious M-power), and samples were immersed in the PBS medium. At a specific time interval, the specimens were collected, blotted to remove the surface medium, and weighed to record the swollen weight (*W*_wet_). The water swelling ratio was calculated as shown in eq 1.
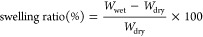
1

The surface water contact angle was measured by a Krüss DSA-100 drop size analyzer. A droplet of the PBS medium (10 μL) was dosed onto the sample surface by using a 22-gauge, blunt-end syringe needle. High-resolution images were captured after 10 s by a built-in digital camera to perform the angle measurements (*n* = 3).

The water vapor transmission rate (WVTR) was measured according to ASTM E96. The dried nanocomposite samples (*n* = 3, thickness: 0.53–0.58 mm) were fixed tightly onto the mouth of a plastic dish containing water by applying a paraffin wax. The dish assembly was then placed at 37 °C with 50% relative humidity in an incubator. The weight loss was recorded on the four-decimal scale, and WVTR was defined by eq 2 as below.
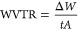
2

Δ*W* (g) is the weight change of the dish assembly, *t* (h) is the time, and *A* (m^2^) is the tested area. WVTR is reported in units of g h^–1^ m^–2^. The calculation was performed where the weight change was steady.

Quasistatic uniaxial tensile tests were performed on a Lloyd LRX, equipped with a 50 N load cell at a strain rate of 50 mm min^–1^ until failure according to ISO 527. The dog-bone specimens (*n* = 6, thickness: 1.19–2.05 mm) of NCHs were prepared by cutting using a mold utensil (Ray-Ran Test Equipment). Tests on the dried nanocomposite samples before saturation in water were also performed (*n* = 6, thickness: 0.64–0.84 mm).

### 2.5 Biodegradability and Biocompatibility Tests

Biodegradability was investigated *in vitro*. Disk-shaped specimens (*n* = 3, diameter: 5.3 mm, and thickness: 0.93–1.82 mm) were sterilized in a 70% ethanol–water solution and dried in a vacuum oven at 37 °C until constant weights were read (*W*_ini_). The specimens were moved into test tubes, each containing 10 mL of the PBS medium with or without 110 U L^–1^ lipase (from the average lipase serum activity in healthy adults of 30–190 U L^–1^).^21^ The test tubes were then placed in a shaker incubator (Stuart SI500) at 37 °C and 100 rpm. The test medium was replaced daily to ensure full enzyme activity during the test period. At a specific time interval, the specimens were collected, washed thrice by a copious amount of water, dried in a vacuum oven at 37 °C overnight, and weighed on the four-decimal scale (*W*_day_). The weight loss by degradation was determined by eq 3.
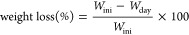
3

Biocompatibility was tested *in vitro* by a cell metabolic assay. Prior to the test, all the disk specimens (diameter: 5.3 mm, thickness: 1.03–1.91 mm) were sterilized in 70% ethanol for 1 day, washed thrice with plenty of water, soaked in DMEM overnight, and placed in an incubator (37 °C, 5% CO_2_). The specimens were then placed in well plates and fixed by sterilized stainless-steel rings. The L929 immortalized dermal mouse fibroblast cell line was chosen according to ISO 10993. Cell trypsinization was performed by trypsin-ethylenediaminetetraacetic acid, followed by neutralization by addition of a warm medium (5 mL). Cells were then collected (centrifugation at 1000 rpm for 5 min), and the optimum cell density of 3.0 × 10^4^ cells per specimen was seeded in DMEM with help of a hemacytometer. Cell-free testing was also done as the negative control. After 1 day of incubation, the old DMEM was removed, and cell-seeded specimens were washed with PBS and moved into new well plates. The resazurin dye solution (0.1 mM in PBS) was added to each well and incubated for 2 h for color change. The colorimetric analysis was performed on a Bio-Tek plate reader at the absorbance wavelength of 570 nm. The test was continued after 4 and 7 days of incubation. The experiments were triplicated. An inverted microscope (Motic AE2000) was used to examine the cellular morphology.

### 2.6 E2 Release Tests

The nanocomposite samples loaded with E2 were prepared by directly dissolving E2 (5 wt %) into the resins before curing at 70 °C for 20 min until homogeneous solutions were acquired. The resins containing E2 were then cured into solid nanocomposite samples in the same manner as previously described. To evaluate the release of E2, nanocomposite disk specimens (*n* = 3, diameter: 5.3 mm, and thickness: 0.90–1.01 mm) loaded with E2 were immersed in 4 mL of PBS. At a specific time interval, 1 mL of the solution was collected and replaced by a fresh PBS medium. The absorption intensity at wavelength λ = 220 nm of the collected PBS containing released E2 was examined under an ultraviolet–visible spectroscope (UV–vis, Agilent Cary 60). A preprepared calibration curve was used to determine the concentration of E2 in the PBS medium (Figure S1).

### 2.7 Malodorous Diamine Control Tests

The control capacity of PUT and CAD with NCHs was determined. NCH disk specimens (*n* = 3, diameter: 5.3 mm, and thickness: 1.12–2.07 mm) were immersed in PBS (3 mL) containing PUT (2.11 mmol) or CAD (0.77 mmol), sealed tightly in a tube, and incubated at 37 °C for 39 h. It should be noted that the initial concentration of PUT and CAD was determined by their average contents in the necrotic tissue of patients with a diabetic foot in the literature.^22^ The incubation time of 39 h was taken from the average time of one wound dressing being applied before it was replaced by another during the treatment of patients.^23^ After incubation, 10 μL of PBS media was collected and the concentration of PUT and CAD in the PBS media was determined by UV–vis spectroscopy utilizing the OPA derivatization reaction coupled with MAA (Supporting Information).^24^ The maximum absorbance (λ_max_) at 487 nm for PUT and 374 nm for CAD was used with calibration curves at different concentrations (Figure S2). PUT- or CAD-only samples without NCHs were also tested and used to normalize the result.

### 2.8 Statistics

All measurements were reported as the mean ± standard deviation with a confidence level of 95%. Error bars indicate standard deviations. One-way and two-way analysis of variance (ANOVA) was performed with the Bonferroni post hoc test by Origin 2024 software.

## 3 Results and Discussion

### 3.1 Preparation and Characterization of NCHs

**Figure 1:**
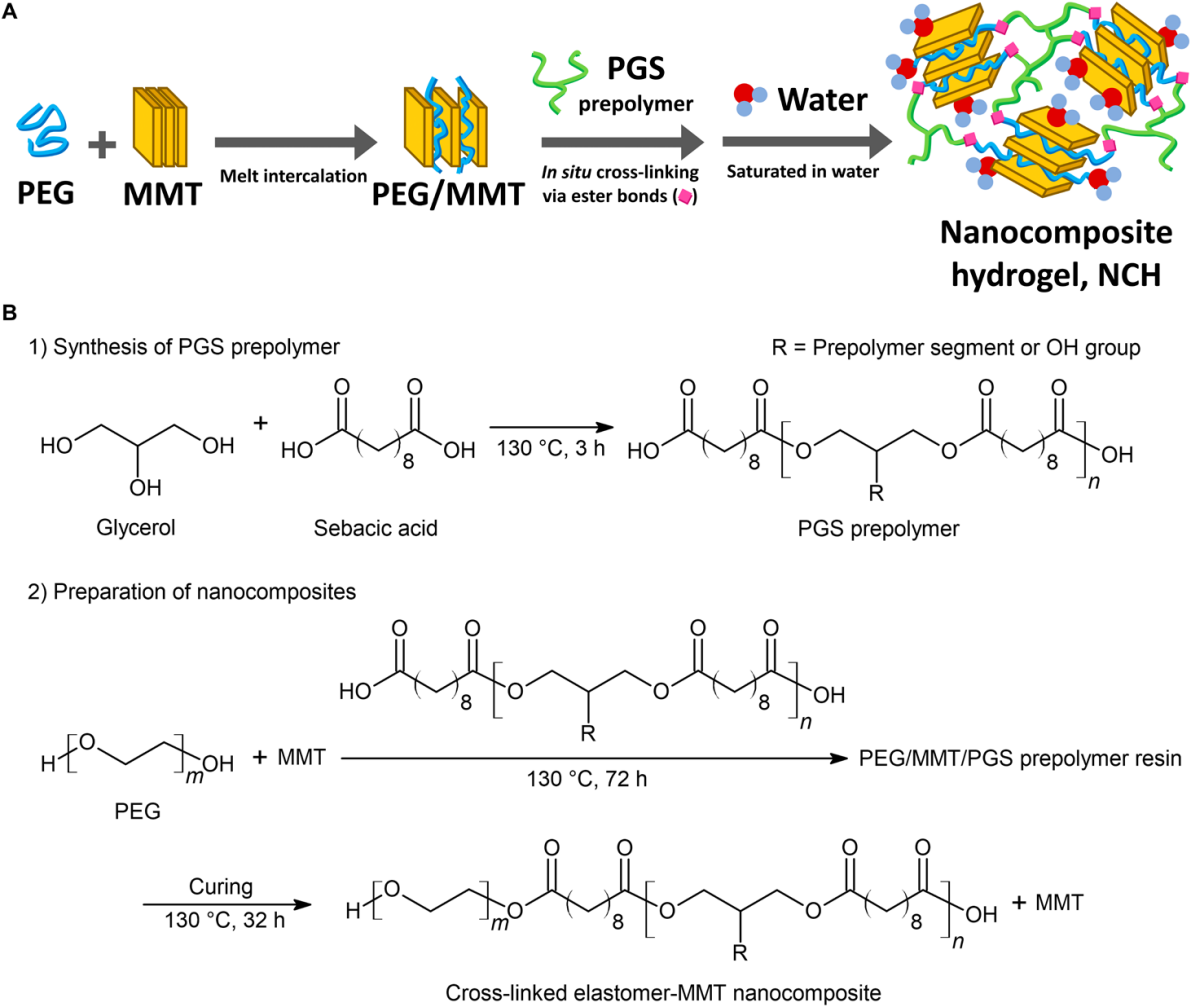
(A) Three steps in the preparation of NCHs: melt intercalation between PEG and MMT to yield PEG/MMT intercalated mixtures, *in situ* cross-linking with the branched PGS prepolymer, and saturation in water to yield NCHs. (B) Scheme showing the reaction between the PGS prepolymer and PEG during preparation of the nanocomposites.

Preparation of NCHs was performed in a combined approach of melt intercalation of PEG into MMT galleries (0, 5, or 10 phr), followed by *in situ* cross-linking between PEG and the PGS prepolymer via ester bonds. The cross-linked nanocomposites were then saturated in water, resulting in NCHs (Figure 1A).

The synthesis of the PGS prepolymer was performed by polycondensation between glycerol and sebacic acid (Figure 1B). It should be noted that the PGS prepolymer in this study was prepared by a relatively short reaction time to achieve a low molecular weight and a nonstoichiometric monomer ratio, which provides additional carboxyl end groups. GPC was performed on the PGS prepolymer. The measured number-average molecular weight (*M̅*_n_), weight-average molecular weight (*M̅*_w_), and polydispersity index (PDI) were 1650 g mol^–1^, 4990 g mol^–1^, and 3.0, respectively. The PDI of over 2 was expected for the branched prepolymer structure and the stoichiometric imbalance between the hydroxyl and carboxyl groups in the synthesis.^19^ FTIR spectroscopy affirmed the successful synthesis of the PGS prepolymer by the peaks attributable to formation of ester bonds (Figure S3). However, the absorption band of carboxyl groups is still shown, confirming that the PGS prepolymer is not fully cured with excessive carboxyl groups to accommodate further reaction.

The melt intercalation of PEG into MMT was then performed to produce the PEG/MMT molten mixtures. PEG and MMT are known to form an intercalated structure, where the polar PEG chain segments are confined within the hydrophilic MMT galleries, expanding the MMT galleries.^25^ This is due to the hydrogen bonding between the MMT surface and PEG, as well as the ion–dipole coordination between PEG and the interlayer cations of MMT.^26^

In the next step, PEG/MMT molten mixtures and the PGS prepolymer were mixed, and the PEG and PGS prepolymer chain segments were cross-linked via a direct esterification reaction at an elevated temperature of 130 °C. The chemical reaction between the PGS prepolymer and PEG is illustrated in Figure 1B. The intercalated PEG in the PEG/MMT mixtures has terminal hydroxyl groups to react with the excess carboxyl end groups in the PGS prepolymer to form ester linkages. This initial esterification was to prepare the viscous PEG/MMT/PGS prepolymer resin products. These resins were then subjected to the final curing step into solid nanocomposite samples, where the remaining hydroxyl and carboxyl groups will continue to react. The branched molecular structure of the PGS prepolymer enabled the formation of the cross-linked polymer network structure, without any additional cross-linking agent. The molar ratio between the organic compounds was 8:13:1 for glycerol:sebacic acid:PEG, giving the stoichiometric ratio between the hydroxyl group and carboxyl group of 1:1 in the final nanocomposite.

To understand whether MMT affects the esterification reaction between the PEG and PGS prepolymer chain segments, the molecular weights of the PEG/MMT/PGS prepolymer resins were analyzed under GPC. These resin samples are, again, the products of the initial esterification reaction before the final curing step.

The *M̅*_n_, *M̅*_w_, and PDI values are listed in Table 1. There was no significant difference in the molecular weights of the two nanocomposite resins containing 5 and 10 phr of MMT; however, slight increases were found in the *M̅*_n_ and *M̅*_w_ of these two samples when compared to the values for the neat polymer resin. This can be due to the catalytic effects of MMT in the esterification reaction.^27^ Nevertheless, MMT does not adversely affect the esterification reaction between the PEG and PGS prepolymer. Although MMT contains hydroxyl groups on its surface (discussed later), the PGS prepolymer is organophilic due to its eight-carbon aliphatic chain in the polymer backbone, which originated from sebacic acid. The organic compound PEG may favorably react with the PGS prepolymer, leaving the inorganic and anionic surface of MMT out of the esterification reaction with the carboxylic groups in the PGS prepolymer.^28^ Moreover, MMT in this study has been modified with excess PEG through intercalation prior to the esterification with the PGS prepolymer. The presence of PEG on the surface of MMT may have sterically hindered the direct chemical interaction between the MMT surface and the relatively large molecule of the PGS prepolymer further.^29^ The PDI values of the PEG/MMT/PGS prepolymers were increased from 3.0 for the PGS prepolymer to 5.46–5.95. These high PDI values were from the increased branching and copolymerization between the PGS prepolymer and PEG.

**Table 1:** Molecular Weights of the PEG/MMT/PGS Prepolymer Resins before the Final Curing Step

	*M̅*_n_	*M̅*_w_	PDI
with 0 phr of MMT	4150	24,710	5.95
with 5 phr of MMT	4570	24,930	5.46
with 10 phr of MMT	4510	25,330	5.62

All the nanocomposites were not dissolved but swollen strongly in various solvents such as acetone, dimethylformamide, dimethyl sulfoxide, ethanol, isopropyl alcohol, and water, affirming covalently cross-linked network structures. This covalent network structure has an advantage in soft tissue engineering and wound healing applications, as physically cross-linked hydrogels could break and disintegrated within the dynamic biochemical environments of trauma sites before the wound is healed completely, causing undesired additional damages on trauma sites.^30^ The amphiphilic swelling characteristic in NCHs is driven by the organophilic PGS and hydrophilic PEG segments, suggesting that NCHs could be postfunctionalized into a multifunctional system that absorbs a desired chemical compound for target biomedical application. Furthermore, the synthesis of NCHs does not involve any surfactants, chemical cross-linking agents, or catalysts. Also, only water is generated as a byproduct during the reaction between the PGS prepolymer and PEG. These are not only beneficial for biocompatibility but also introduce a green synthetic method for medical devices.

There was no significant difference in the residual uncross-linked sol proportions across the NCHs, determined after the extraction by ethanol/water solutions: 22.6 ± 3.4% (0 phr of MMT), 23.7 ± 2.7% (5 phr of MMT), and 22.9 ± 1.7% (10 phr of MMT). This results in the final yields of NCH syntheses of 77.4% (0 phr of MMT), 76.3% (5 phr of MMT), and 77.1% (10 phr of MMT), respectively. No MMT was found in the extract. Together with little to no effects on the molecular weight distribution of the PEG/MMT/PGS prepolymer resins before the final curing step from the GPC study, this extraction test affirms that MMT did not adversely affect the covalent reaction between the PEG and PGS segments in NCHs.

**Figure 2:**
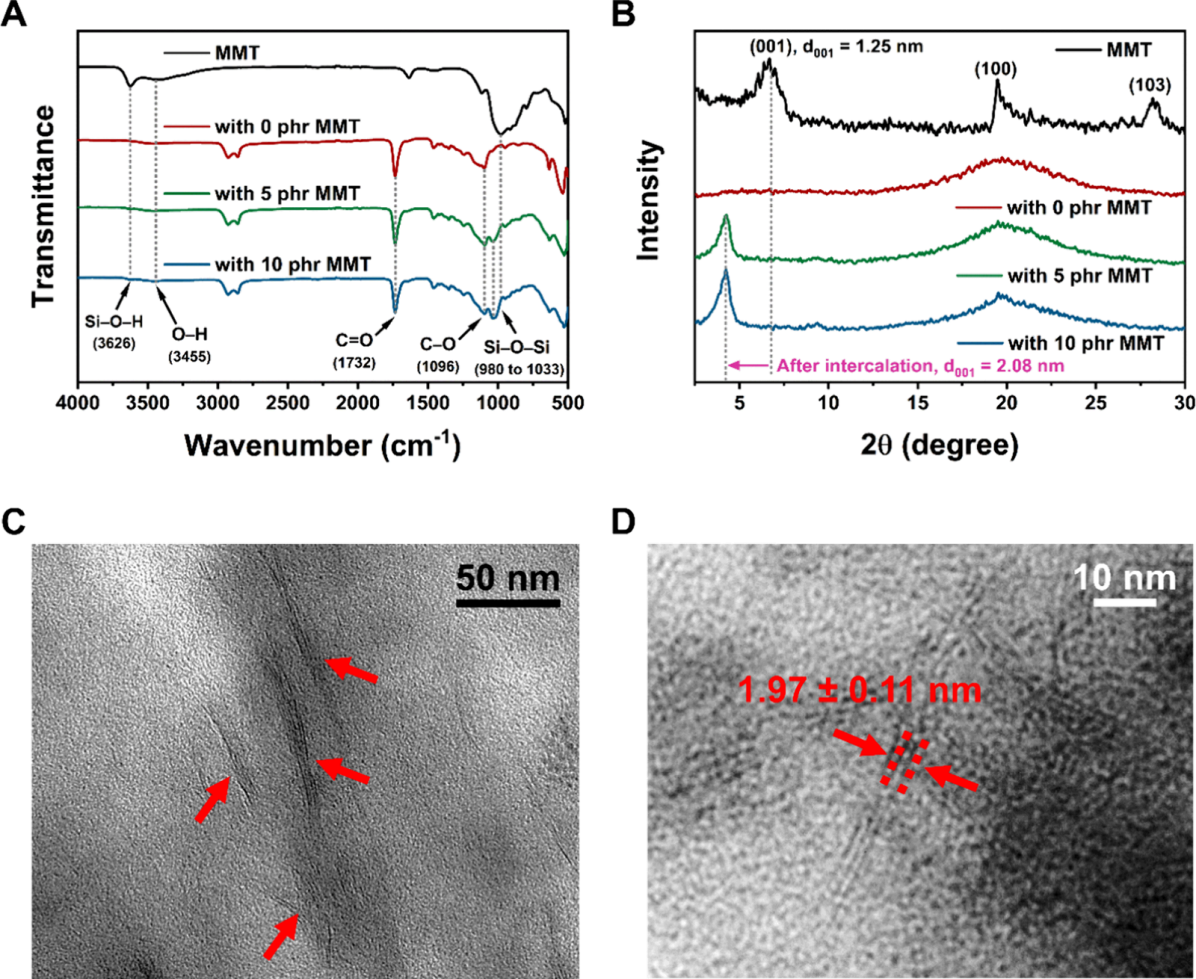
(A) FTIR spectra and (B) XRD patterns of MMT and the nanocomposites with three different MMT contents. (C) TEM micrograph of the nanocomposite with 5 phr of MMT showing an intercalated clay structure (indicated by arrows). (D) High-magnification TEM micrograph of the intercalated clay structure in the nanocomposite.

The chemical structure of the nanocomposites before saturation in PBS was evaluated by FTIR and is shown in Figure 2A. The polymer backbone consisting of ester bonds can be seen by the characteristic ester bond peaks at 1732 cm^–1^ (C=O stretching) and 1096 cm^–1^ (C–O stretching).^31^ The intensity of ester bond peaks does not change with the loading of MMT, confirming again that MMT did not participate in or hinder the ester bonds during the preparation of NCHs. The broad band at 3455 cm^–1^ is from the hydrogen-bonded O–H stretching vibration either by MMT or free hydroxyl functional groups from the polymer segments.^32^ The peaks at 2934 and 2854 cm^–1^ are attributable to the stretching vibration of the methyl groups from the polymer chain. The stretching vibration peak of PEG-related C–O–C can also be seen at 948 cm^–1^.^33^

With the addition of MMT, the most notable changes are the appearances of Si–O–H and Si–O–Si stretching vibration peaks,^34,35^ and the peak intensifies from 5 to 10 phr with the increasing loading of MMT. It should be noted that the Si–O–Si peak blueshifted from the original 980 cm^–1^ in pristine MMT to 1033 cm^–1^ in the nanocomposites. This is attributable to the distorted bond angle of Si–O–Si, caused by molecular confinements from the absorbed PEG within the MMT galleries through hydrogen bonding between the O–H and C–O–C of PEG and Si–O–H and Si–O–Si of MMT.^36^ This strong interaction between the polymer chain segment and MMT in a nanocomposite system can be beneficial for implanted tissue scaffolds and would healing applications, as the MMT bound robustly in the nanocomposite structure would not escape prematurely that may influence the properties of the nanocomposites.^37,38^

The MMT dispersion structure in the nanocomposite samples was analyzed by XRD as shown in Figure 2B. The interlayer spacing (*d*_001_) of pristine MMT used in this study was determined as 1.25 nm (2θ = 7.04°). After the melt intercalation of MMT by PEG to produce the PEG/MMT mixtures, an increased interlayer spacing of 2.11 nm (2θ = 4.18°) was observed (Figure S4). After the final curing step, the interlayer spacing of MMT was 2.08 nm (2θ = 4.24°) in the solid nanocomposite samples. Cross-linking of the PEG chain segments with the PGS prepolymer did not yield significant changes in the MMT interlayer spacing. This confirms that the intercalated structure of PEG and MMT was not significantly altered by cross-linking between PEG and PGS prepolymer segments due to the strong interaction between PEG and MMT.

It can be also seen that the characteristic crystalline PEG peaks at 18.8 and 23.0° under XRD disappeared and were replaced by the amorphous halo at around 2θ = 20° in the cured nanocomposite (Figure S4 and Figure 2B). This is due to the chemical cross-linking of the PEG segment with the branched PGS segments forming a polymer network structure, interfering with the recrystallization of PEG.

The intercalated MMT dispersion structure was also confirmed by a TEM study. Figure 2C shows an intercalated MMT structure in the nanocomposite. A few exfoliated MMT single nanoplatelets can also be seen, but they are minimal. The interlayer spacing of MMT was measured as 1.97 ± 0.11 nm (*n* = 50) in a higher-magnification image (Figure 2D), which is in a good agreement with the previously determined interlayer spacing of 2.08 nm from XRD.

The presence of MMT in the nanocomposite samples was confirmed by a thermogravimetric analysis (TGA) study (Figure S5). The residual weights after the thermal decomposition of organic compounds at 600 °C were 1.7, 6.6, and 11.6% in nanocomposite samples with 0, 5, and 10 phr, respectively, showing good agreement with the MMT content by weight with a sequential increase by 5% between the nanocomposite samples. This suggests that MMT did not escape during the preparation steps, even after extraction with a series of ethanol/water solutions. This affirms that MMT is strongly bound to the polymer matrix. The slightly higher residual weights to the actual MMT contents by 1.6–1.7% in all nanocomposite samples can be attributed to the char formation.^19^ Overall, the nanocomposites consisted of a cross-linked polymer network of the PGS and PEG segments through ester linkages with intercalated MMT by the PEG segment.

**Figure 3:**
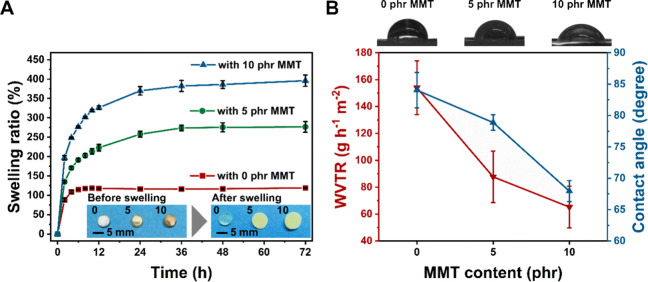
(A) Water swelling ratio of the NCHs with three different MMT contents (0, 5, and 10 phr). The inset photographs show NCHs before (dry nanocomposites) and after saturation in PBS. (B) WVTR and water contact angles of NCHs. The photographs on the top show the shape of water droplets on the surfaces of NCHs.

PGS alone has a minimal water swelling ratio of 2–5%.^13,15^ The hydrophilic PEG and MMT components are responsible for the water swelling properties, resulting in swollen NCHs after saturation in water. The swelling ratio of the NCHs was investigated as shown in Figure 3A.

With the addition of MMT in NCHs, the swelling ratio at equilibrium increased: 119 ± 1% (0 phr), 258 ± 8% (5 phr), and 396 ± 15% (10 phr). The additional hydrophilic and swellable MMT increased the swelling ratio together with osmotic pressure generated by the intercalated PEG segment.^39^ While it took 10 h for NCH with 0 phr to reach the equilibrium in the swelling ratio, NCHs with 5 and 10 phr of MMT took a longer time of 36 h. This delay in reaching equilibrium with MMT is attributable to the fact that clays do not swell instantly but take a series of swelling phases and mechanisms.^40^ Although neat MMT itself can swell in water, MMT in NCHs is not free-swelling. The osmotic pressure of clay must be strong enough to induce the polymer chain relaxation.^41^ This can be further slowed down when the covalently cross-linked PEG and PGS prepolymer segments gain hydrogen bonding interaction with MMT, as MMT now needs to achieve enough osmotic pressure to overcome the relaxation modulus from the covalent polymer network in NCHs that is physically bonded to its surfaces.^42^ The control of swelling properties in the NCHs by MMT plays an important role in wound healing applications by maintaining a moist wound site while absorbing the excessive wound exudates.^38,43^ An adequately moist environment also allows the migration of growth factors and cells for an optimized healing process for wound healing and tissue engineering.^38,44^ The controllable swelling properties can also be utilized to design a swelling-controlled drug release devices as it dictates the diffusion of active molecules or drugs from the hydrogel.^45^

After an injury, the water loss from the trauma site through evaporation is approximately 20 times greater than that of the normal skin surfaces. When a wound is exposed to air, the wound site dehydrates and a scab is formed. This is the body’s response to protect the site from bacterial infection. By doing so, however, the cells in a dry microenvironment will die and the tissue will lose its ability to heal substantially.^46^ Therefore, retaining an adequate amount of moisture is important to optimize the healing process. This ability depends on WVTR, a measurement of water vapor that passes through a unit area during a fixed time period. A too high WVTR may result in dehydration, which not only delays the healing process but also gives a scar tissue.^47^ A too low WVTR may lead to an undesirable accumulation of wound exudates, which often leads to the bacterial infection and malodor, as well as discomfort of patients. To understand the potential applications of NCHs in wound healing and soft tissue engineering, the WVTR of the NCHs was investigated as shown in Figure 3B.

The measured WVTRs in NCHs were 154 ± 20 (0 phr), 87 ± 19 (5 phr), and 65 ± 15 (10 phr) g h^–1^ m^–2^, inversely proportional to the increasing loading of MMT. This is mainly due to the barrier effect of clays, where water vapor diffusion takes a tortuous path in the presence of the intercalated MMT layers in NCHs.^48^ A wound dressing with a specific WVTR must be chosen based on the wound specification, ranging between 84.5 and 29.2 g h^–1^ m^–2^ for optimal proliferation of cells and the healing process.^46,49^ The WVTR of NCHs could be controlled to lie around or within this range with the addition of 5 and 10 phr of MMT.

The surface water contact angle was measured as 84.0 ± 2.8° (0 phr of MMT), 78.9 ± 1.2° (5 phr of MMT), and 68.0 ± 1.7° (10 phr of MMT) (Figure 3B), rendering better surface wettability with the increasing loading of MMT. The presence of surface hydrophilic MMT may have induced this decrease in the water contact angle. The addition of MMT is known to lower the surface tension in nanocomposites, which improves surface wettability.^19^ This controllable surface hydrophilicity is beneficial in wound healing applications as it dictates adsorption of wound exudates to optimize the healing process.^50,51^ Additionally, cells are known to adhere better onto the hydrophilic surfaces, making the NCHs in this study as an attractive candidate in medicine where the cell transfer and culture are involved such as tissue engineering.^52^

### 3.2 Biomimetic Mechanical Behaviors

**Figure 4:**
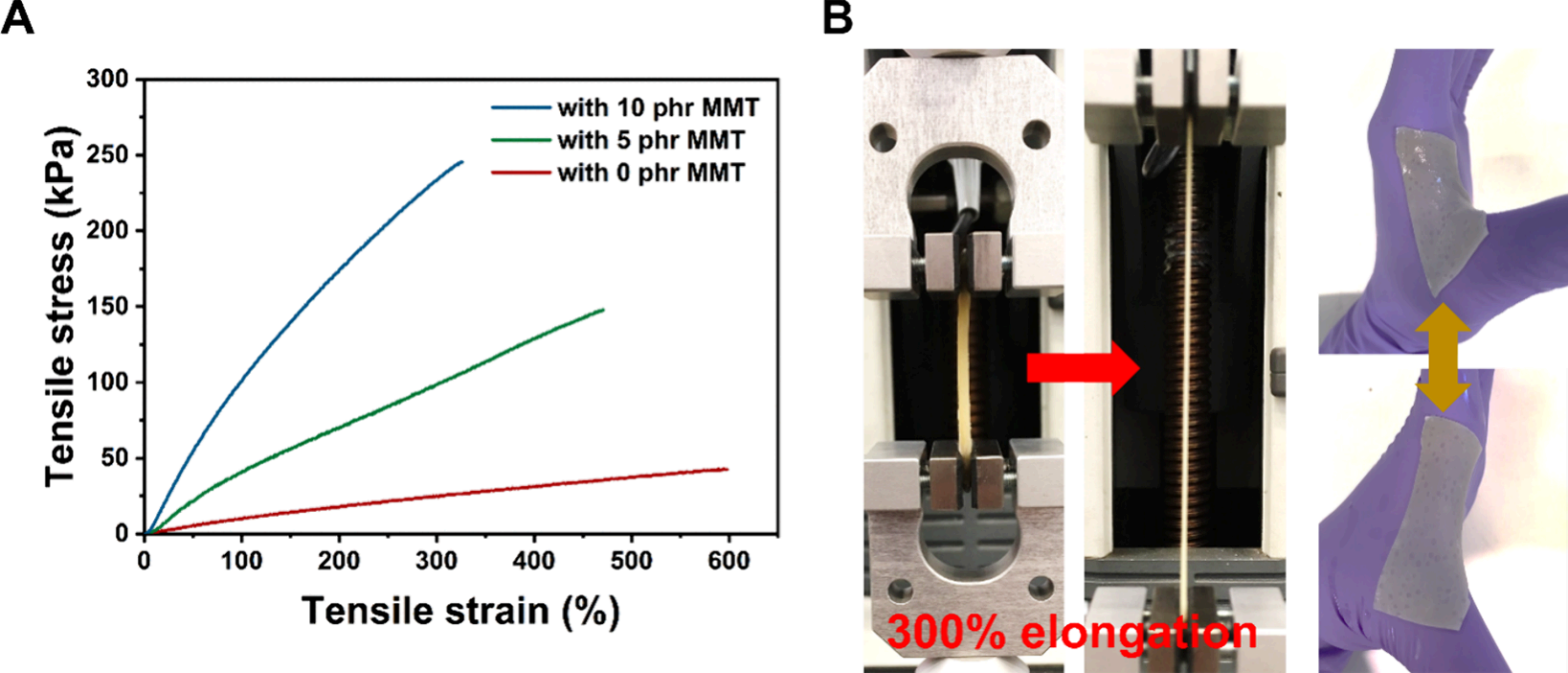
Tensile properties of the NCHs. (A) Representative tensile stress–strain curves of the NCHs. (B) Demonstration of stretchability of the NCH containing 10 phr of MMT during a tensile test and the same NCH placed on a hand joint withstanding a full stretch of the joint without failure.

The mechanical properties of the NCHs were investigated by tensile tests. Representative tensile stress–strain curves are shown in Figure 4A. The tensile parameters are given in Table 2. After the tensile test, all of the NCH samples showed full shape recovery (Figure S6), demonstrating their good resilience. By increasing MMT contents from 0 to 10 phr in NCHs, stiffening, strengthening, and toughening effects in NCHs were found even though the water content becomes higher with the increasing loading of MMT. The Young’s modulus, ultimate tensile strength, and energy to break increased with the increasing loading of MMT while decreasing the elongation at break, suggesting that the cross-linking degree is increased with the addition of MMT. This can be due to the physical interaction between PEG and MMT as found in FTIR through hydrogen bonding. The stiffening and toughening with the increasing loading of MMT can be further attributed to the reinforcement by MMT, in which the load transferred from the polymer matrices to the rigid MMT with a high aspect ratio and large surface area in the nanocomposites.^53^ Strong interactions, such as hydrogen bonding, between the polymer matrices and MMT are crucial in this load transfer.^19^ The mechanical properties of the NCHs may be further modified by changing the stoichiometric balance in the synthesis. For example, softer and more flexible NCH may be prepared by unbalancing the ratio between the hydroxyl and carboxyl groups.

**Table 2:** Summary of Tensile Properties of the Nanocomposite Hydrogels

	**MMT content (phr)**	**Young’s modulus***E***(kPa)**	**ultimate tensile strength σ**_**max**_**(kPa)**	**elongation at break ε**_**tb**_**(%)**	**energy at break***T*(kJ m^–3^)
hydrogel	0	12.6 ± 3.3	37.1 ± 8.0	560 ± 38	125.8 ± 13.5
	5	44.6 ± 2.9	120.4 ± 19.6	412 ± 75	338.5 ± 28.6
	10	105.2 ± 6.0	228.5 ± 15.1	299 ± 45	447.8 ± 38.7
dry	0	40.1 ± 5.8	114.1 ± 5.1	417 ± 43	248.5 ± 11.5
	5	258.0 ± 9.3	502.0 ± 28.6	240 ± 15	664.6 ± 51.4
	10	483.8 ± 28.8	970.4 ± 28.0	172 ± 7	747.4 ± 69.2

Although the elongation at break decreases with the increasing loading of MMT, the NCHs exhibited a nearly 300% elongation at break even with the highest loading of 10 phr of MMT. This is well beyond the range of mechanical strains of soft tissues, which is typically around 15%.^54^Figure 4B demonstrates the good stretchability of the NCH with 10 phr of MMT at a 300% strain and conformability to the bodily movement of the nanocomposites on a curved body joint.

Moreover, the Young’s modulus of NCHs is in the range of various soft tissues such as the myocardium (20–500 kPa), vascular (10–40 kPa), and adipose (30–32 kPa) tissues.^21,55,56^ This achievement of biomimetic mechanical properties is an important material design criterion in soft tissue applications for patients’ comfort and adequate mechanical support during the healing process, as well as to maximize the tissue regeneration as the cellular behaviors are dictated by their surrounding mechanical environment.

Additionally, it is important for a wound dressing not to lose its mechanical integrity while undergoing replacement; otherwise, it can lead to undesirable breakage or eventual dissolution before complete tissue healing or regeneration. As shown in Figure S7, the NCH with 10 phr of MMT shows no evidence of mechanical failure and debris on the applied surface during the detachment.

For the dried nanocomposite samples before water saturation, similar strengthening and toughening effects, together with compromised stretchability, were found with an increasing loading of MMT (Figure S8 and Table 2). These dried nanocomposites have higher Young’s modulus and ultimate tensile strength but lower elongation at break values, when compared to their hydrogel counterparts. This is mainly due to water plasticization in the hydrogels, which disrupts the hydrogen bonding between PEG and MMT, and increases the free volume in the hydrogels, making the polymer chain segments and MMT nanosheets more mobile and the resulting hydrogels more ductile.^57^

**Figure 5:**
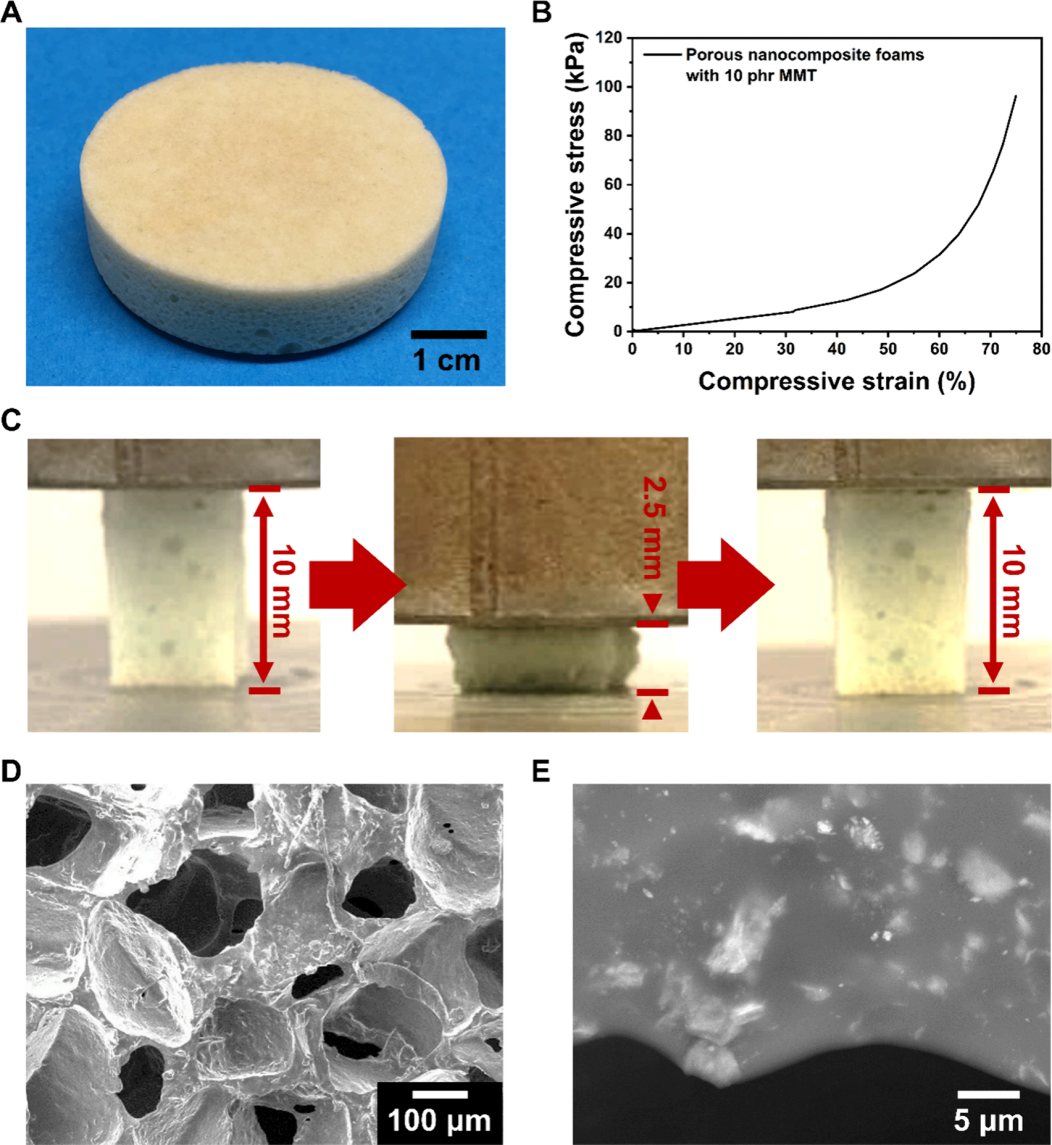
Morphologies and mechanical behaviors of the proof-of-concept porous foam prepared with a nanocomposite sample with 10 phr of MMT. (A) Photograph showing a macroscopic view of the fabricated foam structure. (B) Representative compressive stress–strain curve of the foam until a 75% compressive strain is reached. (C) Foam showing a full and immediate shape recovery after a 75% compressive strain. (D) Interconnected micropore structure shown in a SEM image with a secondary electron detector. (E) SEM micrograph with a backscattered electron detector showing the embedded MMT platelets well-distributed on the wall of a pore.

The PEG/MMT/PGS prepolymer resins of this NCH system before the final curing step are meltable, soluble/dispersible, and so moldable, opening a possibility to build a complex structure. As a proof of concept, a porous foam structure was fabricated for potential applications in soft tissue engineering and wound healing (see the Supporting Information for detailed fabrication and characterization methods) (Figure 5A). For demonstration purposes, a cylindrical shape was chosen, but the shape and size can be controlled based on the mold.

A representative compressive stress–strain curve is shown in Figure 5B, with the compressive Young’s modulus and compressive stress at a 75% strain of 260 ± 40 and 111 ± 20 kPa, respectively. The fabricated porous foam is highly resilient, exhibiting a full and instant shape recovery during mechanical deformation, as shown in Figure 5C and Movie S1.

Under a microscope, pores are interconnected, which is beneficial for higher cellular penetration and proliferation as well as the transportation of nutrients and other chemical species (Figure 5D).^58^ The porosity was calculated as 79 ± 4%, based on the ratio of the density between the porous foam and the nonporous solid sample. The porosity can be tuned based on the ratio between the nanocomposite and salt porogen (Supporting Information). A SEM micrograph with a backscattered electron detector shows that the embedded MMT platelets are distributed well on the walls of the porous scaffolds (Figure 5E). This porous foam structure, biomimetic mechanical property, good biodegradability, and biocompatibility, as well as controlled drug delivery and diamine-controlling functionalities (discussed later), make the NCHs an attractive candidate for soft tissue scaffolds or foam-type wound dressings.

The meltable and soluble/dispersible characteristics of PEG/MMT/PGS prepolymers in our NCH synthesis can be further exploited by state-of-the-art manufacturing routes in tissue engineering application. For instance, fused deposition modeling,^59^ electrohydrodynamics,^60^ or gyration spinning can be applied.^61^ A supporting material would be required during the curing step, which may be sacrificed after shape formation.^62^

### 3.3 *In Vitro* Biodegradability and Biocompatibility

**Figure 6:**
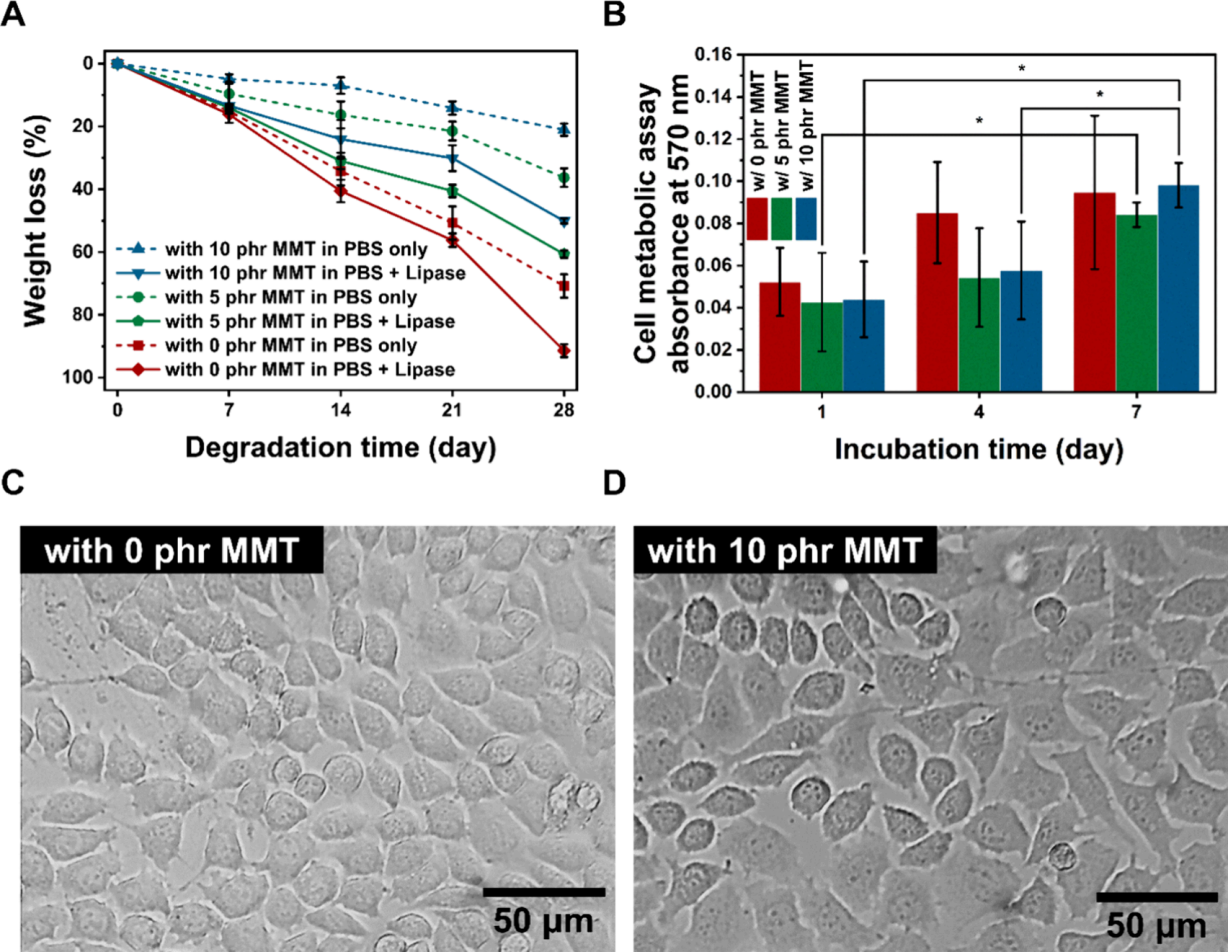
(A) Percentage weight loss in the biodegradation test *in vitro*, showing different degradation rates by MMT contents in NCHs. (B) *In vitro* cell metabolic assay results of L929 fibroblast cells cultured on the surface of NCHs after subtracting the data for cell-free tests. The degree of significance was indicated with the number of asterisks; ***p ≤ 0.001, **p ≤ 0.01, and *p ≤ 0.05. Micrographs show the morphologies of L929 fibroblast cells cultured on the (C) NCH with 0 phr of MMT and (D) NCH with 10 phr of MMT for 7 days.

The effect of MMT on the biodegradability in NCHs was investigated *in vitro*. Two different PBS media were used with or without a lipase enzyme to catalyze the degradation of ester bonds by hydrolysis. Figure 6A shows the percentage weight loss by degradation for 28 days. Without the lipase enzyme, the percentile weight losses after 28 days of degradation were 70.8 ± 3.7% (0 phr of MMT), 36.3 ± 3.0% (5 phr of MMT), and 21.0 ± 1.9% (10 phr of MMT). The degradation of NCHs was faster with the lipase enzyme, resulting in the weight losses after 28 days of 91.4 ± 2.0, 60.6 ± 1.2, and 50.1 ± 0.8%, for 0, 5, and 10 phr of MMT, respectively. When PGS is used alone, the rapid degradation in PGS (as little as 4 days *in vitro*) often limits its use in tissues that require a long-term healing process.^14^ In PGS-*co*-PEG hydrogels, higher degradation rates were found with an increased hydration ability both *in vivo* and *in vitro*, mainly due to the increased diffusion kinetics.^63^ Contrarily in our NCHs, degradation was slower even when the level of hydration was increased with the loading of MMT as discussed previously. The results suggest that the degradation of ester bonds between the PEG and PGS segments as well as within the PGS segments was interfered with by the presence of MMT. The formation of hydrogen bonds between PEG and MMT and the barrier effect from MMT, which, as discussed earlier, contribute to the slower degradation in the NCHs. The controllable degradation behavior found in NCHs with MMT is beneficial to match the degradation requirement of tissue scaffolds to match it with the bodily tissue regeneration rate. Furthermore, it can be also utilized in degradation-controlled drug delivery devices, where the performance is directly dictated by the degradation rate of materials.^64^

The biocompatibility was investigated by the cell metabolic assay *in vitro* as shown in Figure 6B. For all the NCHs, the cell metabolic activity showed steady growth, with the maximum cell metabolic activity on day 7. Two-way ANOVA tests revealed that there was no statistical significance in the cell metabolic activity between NCHs with different MMT contents. One-way ANOVA found statistically significant increases in NCH with 5 phr of MMT between day 1 and day 7. In NCH with 10 phr, the increases between days 1 and 4, as well as days 1 and 7, are found to be statistically significant. This suggests that fibroblast cells were growing rapidly on the NCH samples with 5 and 10 phr of MMT, whereas the growth was steadier on NCH with 0 phr of MMT, without showing any significant increases between culture days.

The cell morphologies are examined on NCHs with 0 and 10 phr of MMT (Figure 6C,D). The cells on both cases appeared normal with adherent cell morphologies. Actin stress fibers can be seen on the pictures, indicating that the fibroblasts are attached well on the NCH surfaces. In both cases, the cells were highly confluent. Overall, no evidence of cytotoxicity was found in NCHs with the increasing cell metabolic activity and the good cell morphologies.

### 3.4 Sustained Drug Delivery and Malodorous Diamine Control

The sustained drug delivery of NCHs was studied. E2 was chosen with its well-known ability to induce angiogenesis as well as cost effectiveness when compared to other proangiogenic agents.^7^ It should be noted that the E2 was loaded into the NCHs before the final curing step at 130 °C, in which the chemical modification of E2 such as oxidation at this elevated temperature can be avoided through the thermal stress. However, E2 is known to be stable at 160–180 °C,^65^ with the main decomposition process taking place in the temperature range of 187–324 °C.^66^ In our TGA study of E2, the degradation onset temperature at 264 °C was found after the initial water loss (Figure S9), which is well above the curing temperature of NCHs of 130 °C. Therefore, it can be assumed that E2 was subjected to little or negligible chemical modifications during the final curing step of NCHs. Moreover, the direct esterification of hydroxyl groups on E2 with carboxyl groups from the PGS prepolymer was ruled out due to the low nucleophilicity and steric hindrance of the phenolic and secondary hydroxyl groups in E2.

**Figure 7:**
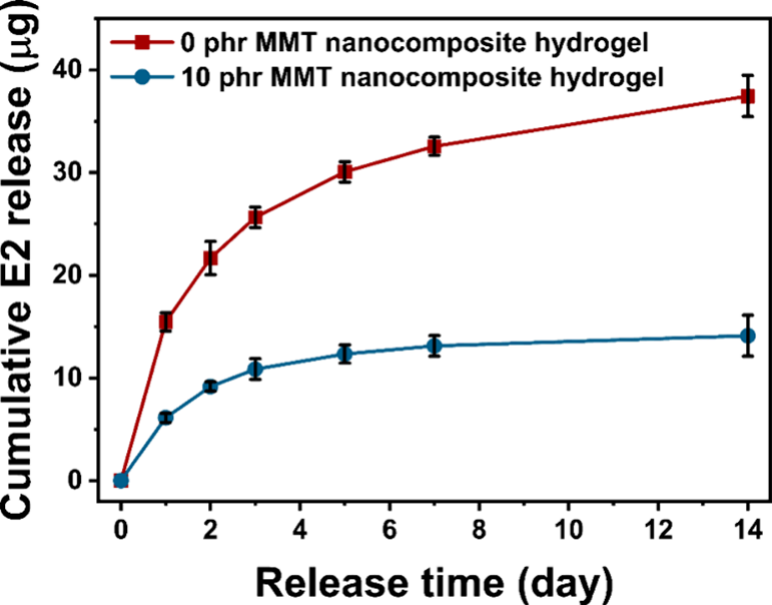
Cumulative drug release profile of E2 with or without 10 phr of MMT in NCHs.

Figure 7 shows the cumulative E2 release profile from the NCHs with 0 and 10 phr of MMT. In both cases, the initial burst release can be seen up to 2 days. In the case of the NCH with 0 phr of MMT, the cumulative E2 release reaches 37.4 ± 2.0 μg in 14 days. However, with the addition of MMT, the release kinetics decreased with a lower slope in the release curve after the initial burst, reaching only 14.1 ± 2.0 μg in 14 days, showing more sustained drug release. This is because the dispersed MMT in the NCH acted as a physical barrier, slowing down the diffusion of the E2 to the releasing medium. With 0 phr of MMT, the high amount of released E2 found in this study can be too high to promote angiogenesis.^67,68^ Thus, the result suggests that the sustained drug delivery achieved with the NCH containing MMT can be utilized to administrate the amount of the proangiogenic agent to promote angiogenesis, which is crucial for the tissue regeneration and healing process.

The high specific surface area (15–50 m^2^ g^–1^), together with the high cation exchange capacity of MMT with its negative electrostatic surface charge, can be utilized to control the malodorous diamines such as PUT and CAD,^69^ which are found in the necrotic tissues from breakdown of amino acids. The excessive generation of PUT and CAD on the trauma sites not only disturbs patients’ comfort with the malignant odors but also adversely affects the tissue regeneration.^47^ For instance, a decreased putrescene level is one of the conditions for cells to migrate across the wounds during the healing process.^12^ These malodorous diamines are positively charged due to the ionization of amine end groups. Therefore, the attractive Coulomb interactions between MMT with a negative surface charge and cationic diamines can effectively control the concentration of diamines in the surrounding environment of NCHs.

**Figure 8:**
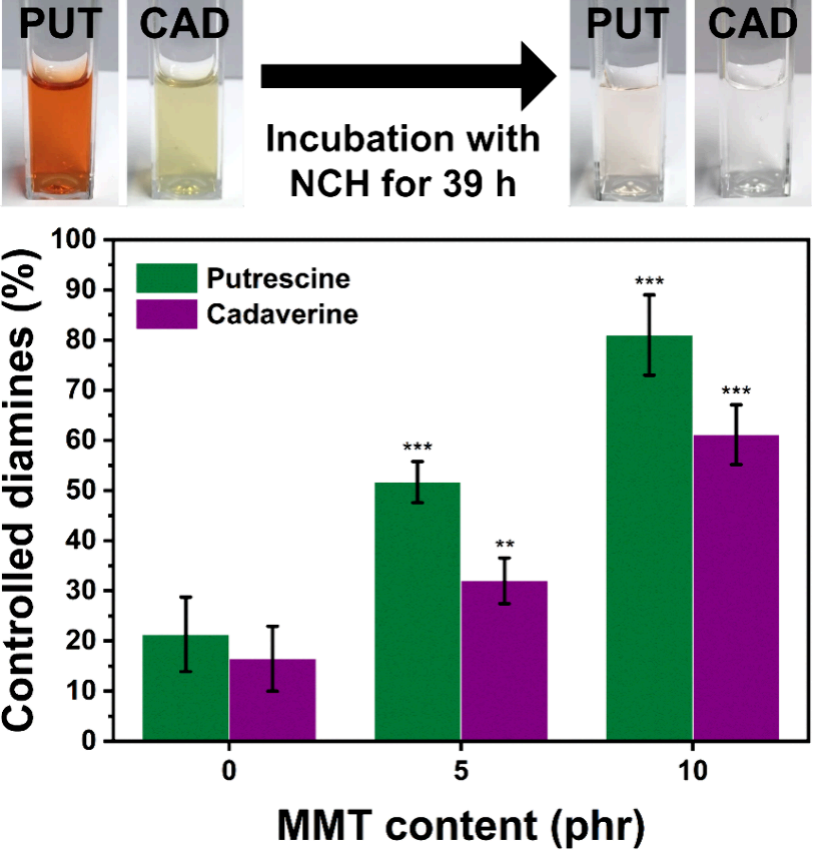
PUT and CAD were controlled by NCHs with different MMT contents after 39 h of incubation. The degree of significance was indicated with the number of asterisks; ***p ≤ 0.001, **p ≤ 0.01, and *p ≤ 0.05. The photographs show the colors of PUT (orange) and CAD (yellow) solutions before and after the incubation with NCH with 10 phr of MMT.

Figure 8 shows control of malodorous diamine compounds of PUT and CAD by NCHs. After 39 h of incubation with NCHs, the concentration of PUT in the solution decreased by 21.3 ± 7.4% (with 0 phr of MMT), 51.7 ± 4.1% (with 5 phr of MMT), and 81.0 ± 8.0% (with 10 phr of MMT). In the case of the removal capacity of CAD by NCHs, they were 16.4 ± 6.5% (with 0 phr of MMT), 32.0 ± 4.6% (with 5 phr of MMT), and 61.1 ± 5.9% (with 10 phr of MMT). In the photographs in Figure 8, the strong orange and yellow colors of PUT and CAD solutions became nearly colorless after incubation for 39 h, with NCH containing 10 phr of MMT (Figure S10). It should be noted that the PUT and CAD solutions are originally colorless. The colors of the solutions were from colorimetric determination with OPA and MAA. The stronger the color, the higher the concentration of PUT and CAD.

One-way ANOVA revealed that differences in the diamine-controlling ability between the NCHs with different MMT contents are statistically significant, indicating that the addition of MMT is an effective method to improve the diamine removal capacity in NCHs. The lower controlling capacity of CAD than PUT by NCHs can be attributed to the higher molecular weight of CAD compared to PUT. This ability to control the diamine products with MMT in NCHs can be studied further in the future, together with soft tissue engineering and wound healing applications.

## 4 Conclusions

A resilient polymer–clay nanocomposite hydrogel system with PEG, PGS, and MMT was developed. The preparation was performed in a combined approach of melt intercalation and *in situ* polymerization with three different MMT contents. PEG was first intercalated into MMT. The PGS prepolymer cross-linked PEG into a polymer network structure through ester bonds, followed by saturation in water resulting in swollen hydrogels. The chemical structure was confirmed by FTIR. The intercalated MMT dispersion structure was confirmed under XRD and TEM.

The surface water contact angle, swelling ratio, and WVTR can be tuned with a tailored amount of MMT, measured as 84.0–68.0°, 119–396%, and 84.5–29.2 g h^–1^ m^–2^, respectively. The tensile properties were also tailored to achieve resilient and biomimetic mechanical behaviors, showing higher modulus and strength, with the addition of MMT due to absorption of PEG into MMT through an intercalated nanocomposite structure, resulting in the physical interaction between PEG and MMT. The Young’s modulus, ultimate tensile strength, and elongation at break were 12.6–105.2 kPa, 37.1–228.5 kPa, and 560–299%, respectively. Good flexibility, stretchability, and full shape recovery after mechanical deformation were found in both hydrogel and a proof-of-concept porous foam structure. For the foam, it was found to have an interconnected pore structure, demonstrating the potential applications in soft tissue scaffolds and wound healing.

The addition of MMT on the biodegradability was also investigated *in vitro*, showing a highly tunable degradation kinetics with MMT. No evidence in cytotoxicity was found in the cell metabolic assay with L929 fibroblast cells, with the increasing cell metabolic activity as well as the normal and confluent cell morphologies discovered under a microscopy study.

The drug release behavior of the NCHs was also examined with a proangiogenic agent, E2. The addition of MMT introduced a sustained release behavior of E2, potentially beneficial to proangiogenic activity, optimizing tissue regeneration. Moreover, the addition of MMT also endows the NCHs the ability to control the malodorous diamine compounds.

Overall, the loading of MMT into the new hydrogel system can alter the hydration, wettability, vapor transmission, mechanical, and degradation properties. Furthermore, new bioactive properties such as sustained drug delivery and controlled malodorous diamine species were achieved with MMT. Together with its proven biocompatibility and porous foam architecture, this new PEG/PGS/MMT hydrogel system has great potential in soft tissue engineering and wound healing, as well as a sustained drug delivery system and odor controlling at wound sites. Future studies can explore proangiogenic activity and wound healing capacity of this new nanocomposite hydrogel system, as well as *in vivo* application of soft tissue engineering to fully address the potential in soft tissue engineering and wound healing applications.

## Data Availability

The dataset underpinning this article is available on PURE for open access, with a description of this article title and DOI.

## Supporting Information Available

Additional experimental details and methods, as well as additional data of Fourier transform infrared spectroscopy spectrum, X-ray diffraction patterns, thermogravimetric analysis, and tensile test curves, including pictures for demonstrations (PDF)
Full and instant shape recovery of the fabricated foam during mechanical deformation (MP4)
