# Supplementary material for: Biomimetic Elastomer–Clay Nanocomposite Hydrogels with Control of Biological Chemicals for Soft Tissue Engineering and Wound Healing
Source: ACS Appl Bio Mater. 2025 Feb 20;8(3):2492–505. doi: 10.1021/acsabm.4c01944 (PMC11921026; doi:10.1021/acsabm.4c01944)
Supplement: Supplementary file 1 — mt4c01944_si_001.pdf [file mt4c01944_si_001.pdf]

Supporting Information

Biomimetic Elastomer−Clay Nanocomposite Hydrogels with Control of Biological Chemicals for Soft Tissue Engineering and Wound Healing

Sungkwon Yoon[a,b] and Biqiong Chen[a,*]

[a]School of Mechanical and Aerospace Engineering, Queen's University Belfast, Stranmillis Road, Belfast, BT9 5AH, United Kingdom

[b]Department of Materials Science and Engineering, University of Sheffield, Mappin Street, Sheffield, S1 3JD, United Kingdom

*Corresponding author. Email address: b.chen@qub.ac.uk

Purification of montmorillonite clay

A desired amount of montmorillonite (MMT) was dispersed in water (1 w/v %) by sonication for 1 h, followed by mechanical agitation for 24 h and allowing for sediment of large aggregates. The rest of supernatant suspension was transferred into a dialysis bag and placed in distilled water to remove excessive cations. The water was changed thrice daily for 4 days, until the conductivity was measured as 10 μS (initial conductivity measured as 321 ± 1 μS, n = 3). Finally, the purified clays were filtered and dried at 60 °C for 48 h in a vacuum oven until a constant weight was observed.[1]

Determination of E2 concentration

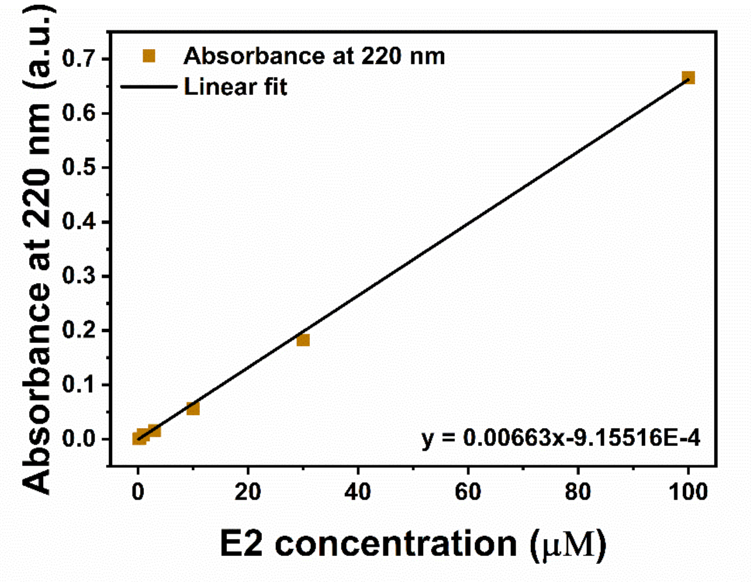

Figure S1. Calibration curve of 17β-estradiol (E2) solution in phosphate buffered saline (PBS) from the standard solutions of E2 prepared at 7 different concentrations (100, 30, 10, 3, 1, 0.3, and 0.1 μM). The stock solution of E2 was prepared first in ethanol at 10 mM and diluted in PBS to achieve the above concentrations.

Putrescene and Cadaverine determination tests

The concentration of putrescene (PUT) and cadaverine (CAD) in the PBS media was determined by utilising the o-phthalaldehyde (OPA) derivatisation reaction coupled with mercaptoacetic acid (MAA).[2] Prior to the test, OPA and MAA were dissolved separately in methanol at 0.2 M and kept at 4 °C for 24 h prior to use. After the incubation of PUT and CAD solutions in PBS media with or without the nanocomposite hydrogel samples, 10 µL of PBS media were collected. The OPA and MAA solutions (20 µL each) were added into the PBS media. The final volume was adjusted to 2.0 mL by PBS. The mixture was then sealed tightly and heated at 60 °C for 12 min using a heating mantle. Colourimetric analysis was performed using an ultraviolet-visible spectroscope (UV-vis, Agilent Cary 60) to determine the concentration of PUT and CAD at the maximum wavelengths of λ_{max} = 487 nm and λ_{max} = 374 nm, respectively, with calibration curves (Figure S2) prepared as below.

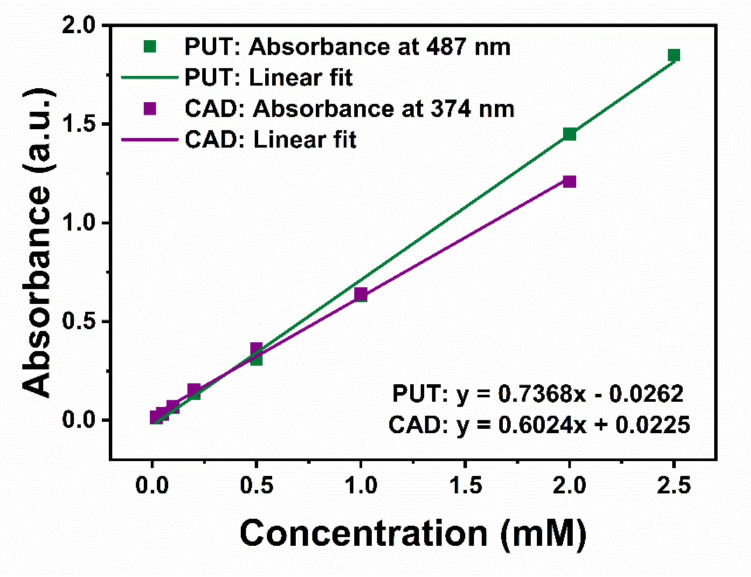

Figure S2. Calibration curves of PUT and CAD acquired under UV-vis spectroscopy. The standard solutions of PUT and CAD were prepared at the eight (PUT; 20 µM, 50 µM, 0.1 mM, 0.2 mM, 0.5 mM, 1.0 mM, 2.0 mM, and 2.5 mM) or seven (CAD; 20 µM, 50 µM, 0.1 mM, 0.2 mM, 0.5 mM, 1.0 mM, and 2.0 mM) different concentrations and used to generate the calibration curves. CAD at 2.5 mM was not tested as it exceeded the upper limit of absorbance that can be detected by the equipment. It should be noted that the concentration range of PUT and CAD in the actual PUT and CAD removal tests with the nanocomposite hydrogel samples was well below the highest concentrations of the calibration curves.

FTIR characterisation of the PGS pre-polymer

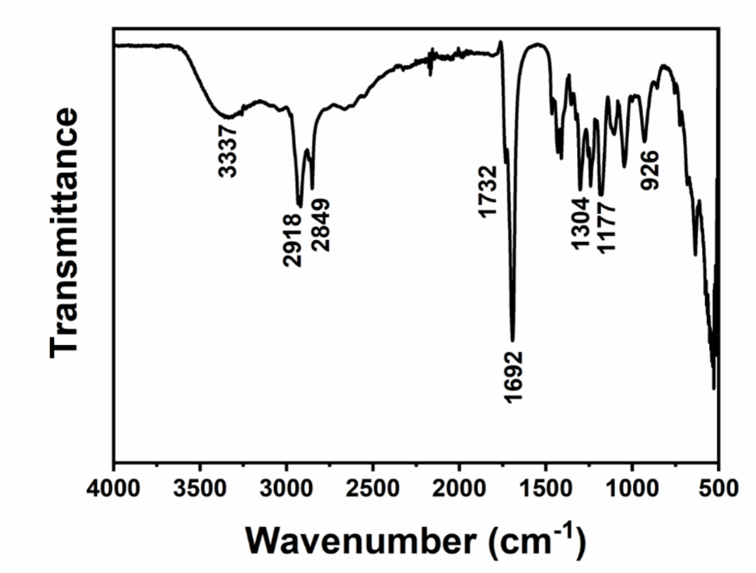

Figure S3. The attenuated total reflectance Fourier transform infrared spectroscopy (FTIR, PerkinElmer Spectrum One NTS analyser) spectrum of the PGS pre-polymer in this study. The broad band between at 3337 cm^{-1} is from hydroxyl groups (O–H).[3,4] Two peaks at 2918 and 2849 cm^{-1} are stretching vibration of methyl groups (C–H).[3,4] The peaks at 1732 cm^{-1} (C=O) and 1177 cm^{-1} (C–O) are the characteristics of ester bonds.[4] Carboxyl group peaks are shown at 1692 cm^{-1} (dimer C=O), 1304 cm^{-1} (C–O stretching), and 926 cm^{-1} (O–H bending).[4]

X-ray diffraction study on MMT and PEG with different MMT contents

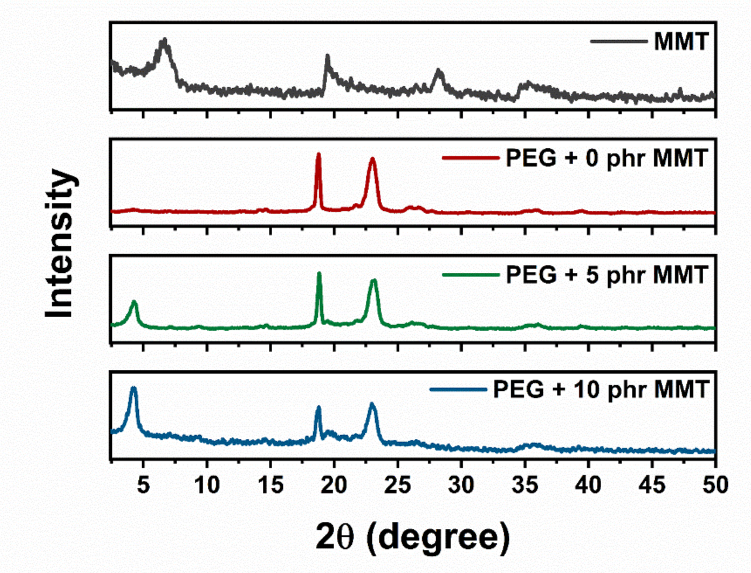

Figure S4. X-ray diffraction patterns of MMT and poly(ethylene glycol) (PEG) with different MMT contents. MMT shows (001) peak at $2\theta = 7.04°$. After the melt intercalation of MMT by PEG, the (001) peak shifted to $2\theta = 4.18°$. The peaks from crystalline characteristics from PEG are also shown from $2\theta = 18.8°$ to $2\theta = 23.0°$.[5]

TGA study on MMT and dry nanocomposite samples

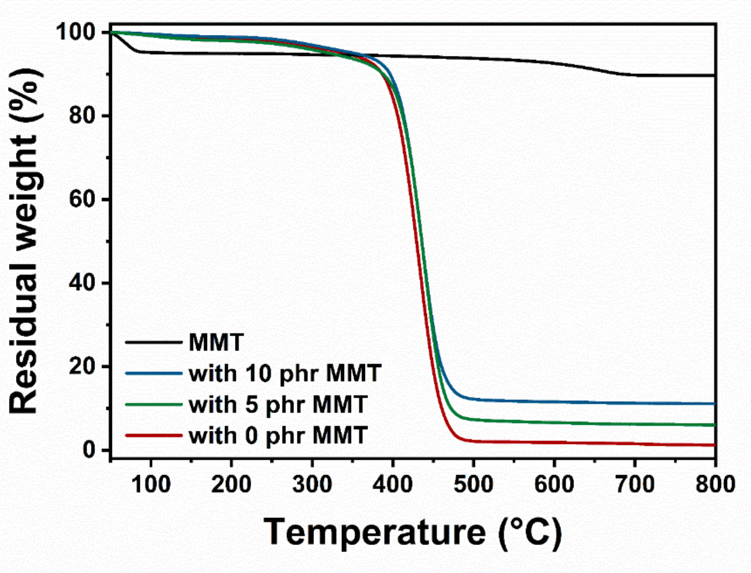

Figure S5. Thermogravimetric analysis (TGA) of MMT and dry nanocomposite samples with three different MMT contents (0, 5, and 10 phr). TGA was performed on the PerkinElmer Pyris 1 TGA, from 50 to 800 °C at 10 °C min^{-1} in a N_2 atmosphere. MMT exhibited two major weight losses: (1) dehydration of free water at 100 °C and interlayer water up to 200 °C and (2) dihydroxylation and heat absorption between 600 and 800 °C.[6] The residual weights of nanocomposites after the thermal decomposition of organic compounds at 600 °C were 1.7% (0 phr MMT), 6.6% (5 phr MMT), and 11.6% (10 phr MMT), respectively.

Demonstration of mechanical properties and resilience of nanocomposites

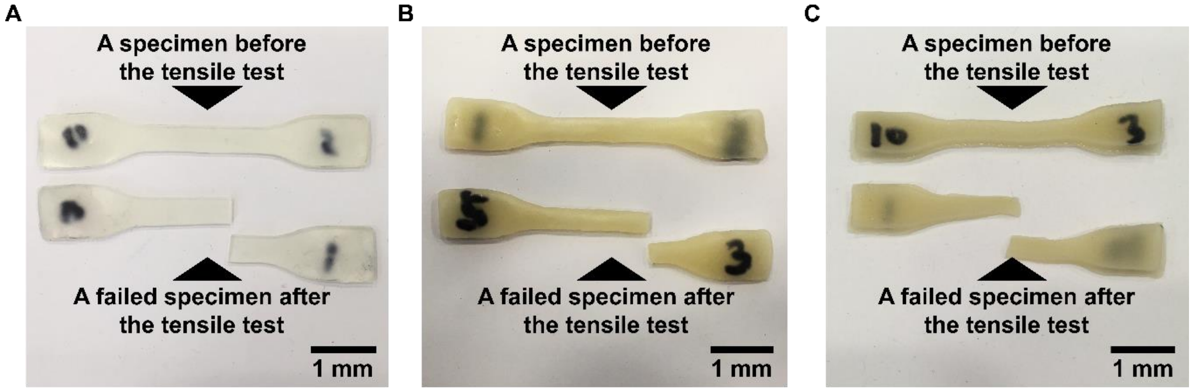

Figure S6. Nanocomposite hydrogel samples immediately after tensile tests, showing full shape recovery: (A) with 0 phr of MMT, (B) with 5 phr of MMT, and (C) with 10 phr of MMT.

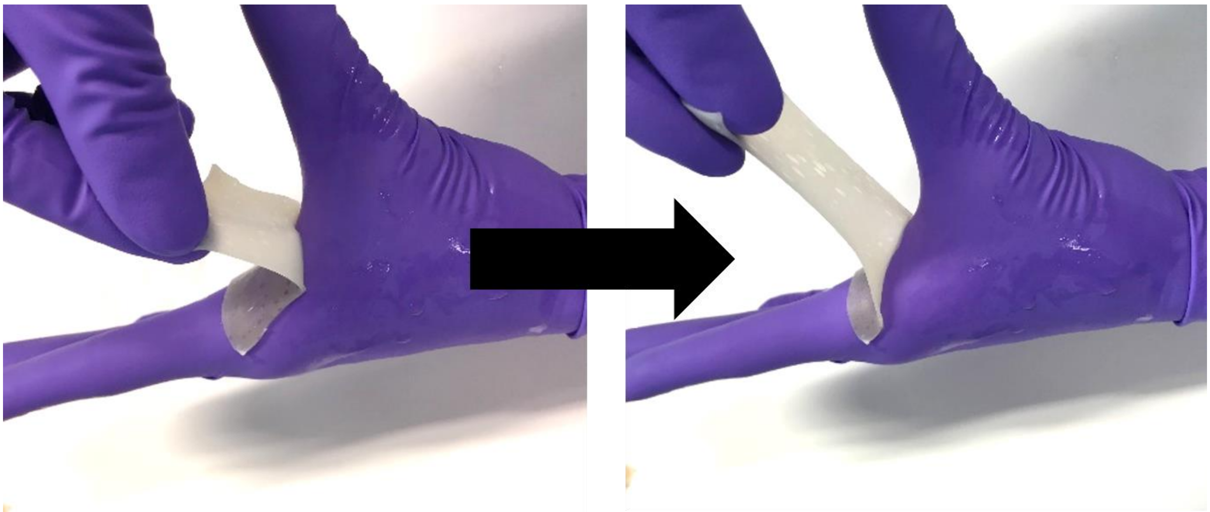

Figure S7. A nanocomposite hydrogel sample with 10 phr of MMT leaves no marks or debris on the surface when being detached.

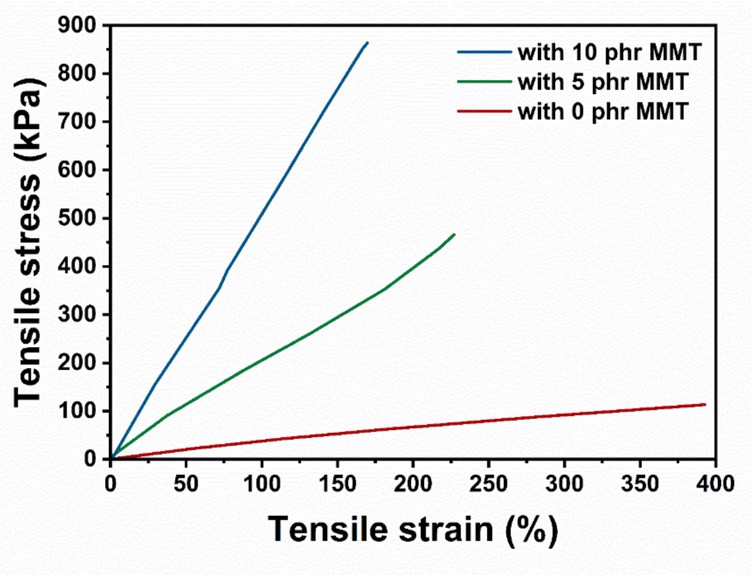

Figure S8. Representative tensile stress-strain curves of dry nanocomposite samples with three different MMT contents (0, 5, and 10 phr) before saturation in water.

Proof-of-concept fabrication and compressive testing of nanocomposite foams

A nanocomposite foam was fabricated by a combined approach of salt-leaching and freeze-drying as a *proof-of-concept*. First, doubly sieved salt particles (300 µm, from a local store) were prepared and added into the molten nanocomposite resin prior to the curing with 10 phr of MMT (salt:resin=3:1 weight ratio). An overhead stirrer at 80 rpm for 20 min was applied to make a mixture. The mixture was then cast into a polytetrafluoroethylene dish and cured in a vacuum oven at 130 °C for 32 h. After curing, the salt particles were leached out by a copious amount of water for 2 days. Subsequently, the sample was subjected to freeze-drying using a FreeZone Triad Cascade Benchtop Freeze Dryer (Labconco, USA). A three-step freeze drying procedure was used: freezing, primary drying, and secondary drying. For the freezing stage, the temperature was reduced from room temperature (21 ± 2 °C) to −50 °C and maintained for 6 h. The primary drying was performed at −10 °C for 24 h under a reduced pressure of 0.1 mbar. The secondary drying stage was conducted at 40 °C for 24 h under the same vacuum pressure. Throughout the freeze-drying stages, the temperature ramping rate was 1 °C min^{-1}.

Compressive tests of the foams were performed on a Lloyd LRX, equipped with a 50 N load cell and a pair of disk compressors. A rate of 50 mm min^{-1} was used till 75% strain. Rectangular test specimens (n = 4) with length of around 5 mm and thickness of around 8 mm were prepared by cutting.

TGA of 17β-estradiol for sustained drug delivery

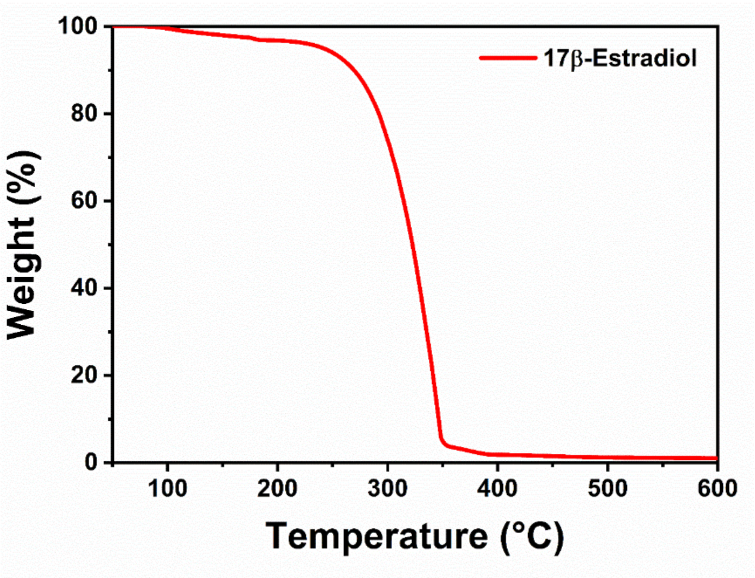

Figure S9. TGA of 17β-estradiol showing the initial water loss up to 176 °C, and the main degradation onset temperature at 264 °C

References

(1) Hong, S.-I.; Rhim, J.-W. Antimicrobial Activity of Organically Modified Nano-Clays. *J Nanosci Nanotechnol* **2008**, *8* (11), 5818–5824. https://doi.org/10.1166/jnn.2008.248.

(2) Qi, X.; Wang, W. F.; Wang, J.; Yang, J. L.; Shi, Y. P. Highly Selective Colorimetric Detection of Putrescine in Fish Products Using O-Phthalaldehyde Derivatization Reaction. *Food Chem* **2018**, *259* (March), 245–250. https://doi.org/10.1016/j.foodchem.2018.03.131.

(3) Frydrych, M.; Chen, B. Large Three-Dimensional Poly(Glycerol Sebacate)-Based Scaffolds – a Freeze-Drying Preparation Approach. *J Mater Chem B* **2013**, *1* (48), 6650–6661. https://doi.org/10.1039/c3tb20842g.

(4) Cai, W.; Liu, L. Shape-Memory Effect of Poly (Glycerol-Sebacate) Elastomer. *Mater Lett* **2008**, *62* (14), 2175–2177. https://doi.org/10.1016/j.matlet.2007.11.042.

(5) Chen, B.; Evans, J. R. G. X-Ray Diffraction Studies and Phase Volume Determinations in Poly(Ethylene Glycol)-Montmorillonite Nanocomposites. *Polym Int* **2005**, *54* (5), 807–813. https://doi.org/10.1002/pi.1774.

(6) Liang, N.; Liu, Y.; Liao, X.; Luo, Z.; Chen, D.; Liu, X.; Zhang, H. Preparation and Characterization of Anion-Exchange Membranes Derived from Poly(Vinylbenzyl Chloride-Co-Styrene) and Intercalated Montmorillonite. *Polym Adv Technol* **2017**, *28* (6), 728–735. https://doi.org/10.1002/pat.3959.
